# How Much Credence Does It Take? Evidence on the Trade-Off between Country-Of-Origin Information and Credence Attributes for Beef from a Choice Experiment in Sweden

**DOI:** 10.3390/foods6100084

**Published:** 2017-09-25

**Authors:** Carl Johan Lagerkvist, Sebastian Hess, Helena Johansson

**Affiliations:** 1 Department of Economics, Swedish University of Agricultural Sciences, Uppsala 75007, Sweden; 2 Institute of Agricultural Economics, Christian-Albrechts-Universität zu Kiel, Kiel 24118, Germany; shess@ae.uni-kiel.de; 3 Lund University, AgriFood Economics Center, Lund 22007, Sweden; Helena.Johansson@agrifood.lu.se

**Keywords:** food labelling, information cues, consumer acceptance, choice experiments

## Abstract

Based on a discrete choice experiment with 336 consumers, this study investigated whether the consumer propensity to choose a simplified European Union (EU) vs. non-EU denomination of origin for beef, instead of a specific country-of-origin (COO) denomination, depends upon the amount and type of credence information provided to the individual. The likelihood of choosing the EU/non-EU denomination of origin depended on the total number of other labelling credence attributes provided and also on the type of detailed credence attributes present in the choice. The presence of cues relating to animal welfare and far-reaching traceability had the highest likelihood of influencing the choice of the EU/non-EU denomination of origin. The compensatory qualities of each credence attribute in relation to the EU/non-EU origin denomination thus differed.

## 1. Introduction

Consumers have difficulties forming expectations of meat quality [1], and there has been an increasing use of labels to provide consumers with information about credence quality attributes such as health-related effects, convenience, ethical factors, farm animal welfare, etc. [2,3]. The importance of country-of-origin (COO) information for beef has been attributed to its role as a proxy, or heuristic, for meat safety through an availability bias related to country associations [4,5], hence companies are working to reduce consumer quality uncertainty and choice complexity. Findings by Dickinson and Bailey [6] confirm that consumer preference for COO might be low if not supported by other safety and credence attributes.

For non-minced beef, following the Bovine spongiform encephalopathy (BSE) crisis, there has been a requirement in the EU since 2000 to provide information about place (i.e., country) of origin (EU 1760/2000), including the presentation of an individual reference or code number referring to the specific animal and a licence number for the slaughterhouse. This mandatory labelling requirement regarding origin was motivated by an information asymmetry perspective in relation to consumer concerns about food safety and quality.

The European Commission has recently extended mandatory origin labelling to cover fresh and frozen pig, poultry, sheep, and goat meat (Regulation (EU) 1169/2011). Compared with the beef system, the labelling scheme for pig/poultry/sheep/goat is less demanding and requires information on the country in which the animal is reared and slaughtered (Commission Implementing Regulation 1337/2013). This extended labelling was prompted by the fact that consumers are mostly interested in the rearing system and that the cost of providing information on birth would be high. A possible labelling alternative for these products is defining origin as an EU/non-EU denomination, without reference to a specific country [7]. This form of labelling is currently employed for honey (Directive 2001/110/EC).

There is extensive literature on COO effects, but the cognitive issues associated with a wider than country-specific denomination of origin are poorly understood. The objective of the present study was therefore to examine how the depth and content of attribute information processing influence the choice of the EU/non-EU denomination for beef among Swedish consumers. Information processing strategies and information searches have a long-standing tradition in consumer behaviour research [8]. The first working hypothesis we tested here was that the propensity to choose the EU/non-EU denomination of origin depends on the total number of other labelling credence attributes so as to compensate for the lack of specific COO information. We also investigated whether the probability of choosing the EU/non-EU denomination of origin depended on interaction effects between the number of quality cues and the price level in order to examine the extent of direct and indirect effects on choice. The second hypothesis related to the detailed type of credence attributes that are sought, in combination with the associated price level, to base the choice of a beef product with the EU/non-EU denomination. This part of the analysis addressed the compensatory qualities of each credence attribute in relation to the new, broader than national type of denomination. By testing the joint hypothesis that all price levels are zero, we then analysed whether and under what circumstances price may not matter at all for information provision. Furthermore, by testing hypotheses about the equality of the coefficients of each discrete price level and the number of information attributes provided, we assessed whether there are cases in which an incremental price or information change does not change the probability of the corresponding choice decision.

## 2. Theoretical Background

### 2.1. Cue-Based Decision Making

Cue-based decision making has recently come to be recognised as a stand-alone evaluation and consumer decision model [9]. This approach integrates cue utilisation (i.e., the search process) with consumer heuristics (i.e., decision making). A cue attribute refers to a product feature with a level (search criteria) that correlates with the levels of other attributes that may be available to reinforce the interpretation of the cue or that correlates with unavailable attributes so that the available cue can act as a proxy for, override, or replace these absent attributes. Consumers are then modelled to make decisions based on immediately present information, which is consistent with positional theory [10] in that they seek to gain information so that they can position their past experience in relation to new information, while asserting their own position (i.e., willingness-to-buy; like or dislike). The role and use of cue attributes are closely related to credence labels. Following Zand [11], credence quality cues can then be expected to be considered ad hoc and only to the extent that they are congruent with current behaviour or deeds. However, when encountered, credence quality cues seldom occur in isolation, but rather with other cues as a related set in a choice situation. Those sets, when and where encountered, provide a temporal structure, which ‘triggers a short-term set of related heuristics’ [9], p. 95.

### 2.2. Information Processing and Use of Labels

Informational theory research into consumers’ information search processes has evolved over three dimensions: (a) the amount of information sought (‘the depth’) [12]; (b) the type of information examined (‘the content’) [8]; and (c) the order or pattern by which information is acquired (‘the sequence’) [13]. In this study, the focus was on the two former dimensions. The order or pattern relates to the accessibility of attribute information in people’s memory, which is influenced by the quantity and frequency at which an individual attends to information but is not directly part of judgement and choice [14,15].

Regarding information depth, the literature suggests that relatively few informational items are used by consumers as a basis for their purchasing decisions and that relevance may be predicted by relatively few pieces of information (e.g., [16]). In this respect, the EU/non-EU denomination and the specific COO may differ in the relative influence of origin information versus other product information. With aspects of familiarity [17], perceptions of quality [18], symbolic or emotional associations [19], and consumer ethnocentrism [20], COO can more specifically summarise consumers’ perception of other product attributes. In contrast, a broader EU/non-EU origin label may require a larger assortment of informational cues for consumers to form an evaluation leading to the choice of such a product. This would be consistent with findings within a meta-analysis by Verlegh and Steenkamp [19], who reported that the link between COO and perceived quality was particularly strong.

The type of information that consumers are likely to consider when choosing an EU/non-EU labelled product instead of a specific COO product is also of interest when analysing the information search process. A broader denomination implies more uncertainty, which might imply that, as found by Chisik [21], it would be reasonable to expect consumers to strive for cognitive consonance, considering informational cues to support the formation of product preferences. Hence, choices for the EU/non-EU denomination may be conditioned by the presence of other labelling attributes that are in line with the person’s attitudes, behaviour, beliefs, and/or knowledge to the extent that these attributes can compensate for the eventual lack of credence quality related to the more uncertain origin denomination.

Price information is similar to other labelling attributes in being subjected to eventual attention and processing by consumers in a choice situation [22]. Lockshin et al. [23] showed that low-involvement consumers were more prone to use price as a criterion in making purchase decisions than were high-involvement consumers. Price can be expected to influence product choices in three ways: in a compensatory way (trade-offs between the importance of the price versus other cues; in a conjunctive way (i.e., price is taken into consideration but only within a certain span); and, finally, as a simplifying choice heuristic [22]. Price information processing therefore relates to the depth and the content of the search process.

The interplay of and meaning in the interpretation of information elements establish a complicated search process, in particular as the preferences for labelling are likely to be diverse due to the existence of quite heterogeneous views on quality [2]. Little research seems to be available concerning the likely importance of labelling information. One exception is the study by Verbeke and Ward [24], in which an ordered probit model was used to assess the impact of individual and labelling characteristics. A meta-analysis by Verlegh and Steenkamp [19] and results from Gao and Schroeder [25] suggest that consumer labelling preferences are highly conditional upon what labelling attributes are/are not included in the survey.

## 3. Material and Methods

### 3.1. Recruitment and Data Collection

The present analysis draws on data collected in Sweden in November to December 2012. The respondents (aged 18 to 75) were randomly recruited from an online panel provided by a marketing research company (*n* = 440). The respondents were initially screened for their beef purchasing frequency (purchasing beef at least one to two times per quarter-year was used as the cut-off). The response rate was 76.4% (*n* = 336). A small participation fee in the form of reward points (equivalent to SEK 10.5) was provided. Detailed demographic characteristics are shown in Table 1. The sample contained slightly more men (54%) than women (46%) (Table 1). The participant age was slightly higher than the corresponding age distribution of the Swedish population.

The respondents were first asked (on a scale from 1 = I look at all to 5 = I do not look at any) about their self-reported attention to beef labelling information. A majority of respondents indicated that they look at all or most of the labelling information when buying beef (Table 2). These results are similar to findings on how often consumers read nutrition fact panels on labels [26].

### 3.2. Stimuli: The Discrete Choice Experiment

Choice experiments (CEs) have become widely used in the field of food choice research. This approach is useful for understanding the demand for a new product with new attributes and also for examining behavioural issues [27]. When carefully designed, a CE conducts a temporal evaluation of attributes over a range of choices in order to reveal any significant relationships between choice and available attributes.

As well as being useful for the analysis of product demand, a CE provides an attractive, yet not widely explored, feature in allowing for the estimation of the choice probability for a product alternative, conditional upon the depth and content of the consideration set available to the individual when being asked to make the choice. In the context of food choice, it has been reported that CE estimates are sensitive to the dimensionality of the experimental design. Gao and Schroeder [25] and Caputo, Scarpa, and Nayga [28] found that the stability of preferences for cue attributes was affected by the number of attributes. They also reported that this effect existed for credence-type attributes but not for so-called independent attributes (i.e., aspects related to the physical nature of the product, information on which can be directly observed by the consumer). These results corroborate findings by Hensher [29] and suggest that the information processing strategy of individuals relates to the functional relationship between attributes available to individuals in the choice situation. Therefore, a partial profile design was created for the CE in this study. The partial profile design, which was first described by Green [30], allows for more realism in the decision-making process, as product comparisons in real-life situations are less likely to always include identical labelling attributes. To replicate this presence/absence of certain information, the attribute levels within our CE were set as binary. In this way, at each choice (the temporal structure), a set of unique labels (i.e., cues) was made available. This aspect of the partial profile design makes the CE more reflective of the cue-based decision-making model by Hamlin [9].

The partial profile design presents choice tasks that vary only in the levels of a subset of all attributes, which distinguishes it from a full-profile design (across all choice tasks and concepts, all attributes are present, although the levels of each attribute vary according to the experimental design). A full-profile design is not representative of more realistic in-store choice situations, as beef products typically differ in the extent of the labels presented on the package. The full-profile design has, however, been widely used in applied research, especially when the number of attributes is not too large or when there are only a few levels per attribute, or both (as recommended by Green and Srinivasan [31]).

The use of a partial profile design is not without problems, however, as previous findings suggest that the importance of the price attribute may be reduced, leading to inflated willingness-to-pay estimates. Based on research by Hensher [29] showing that the likelihood of misspecified estimates increases with a narrower attribute range, it was therefore deemed relevant to include a rather wide array of price levels.

**Figure 1:**
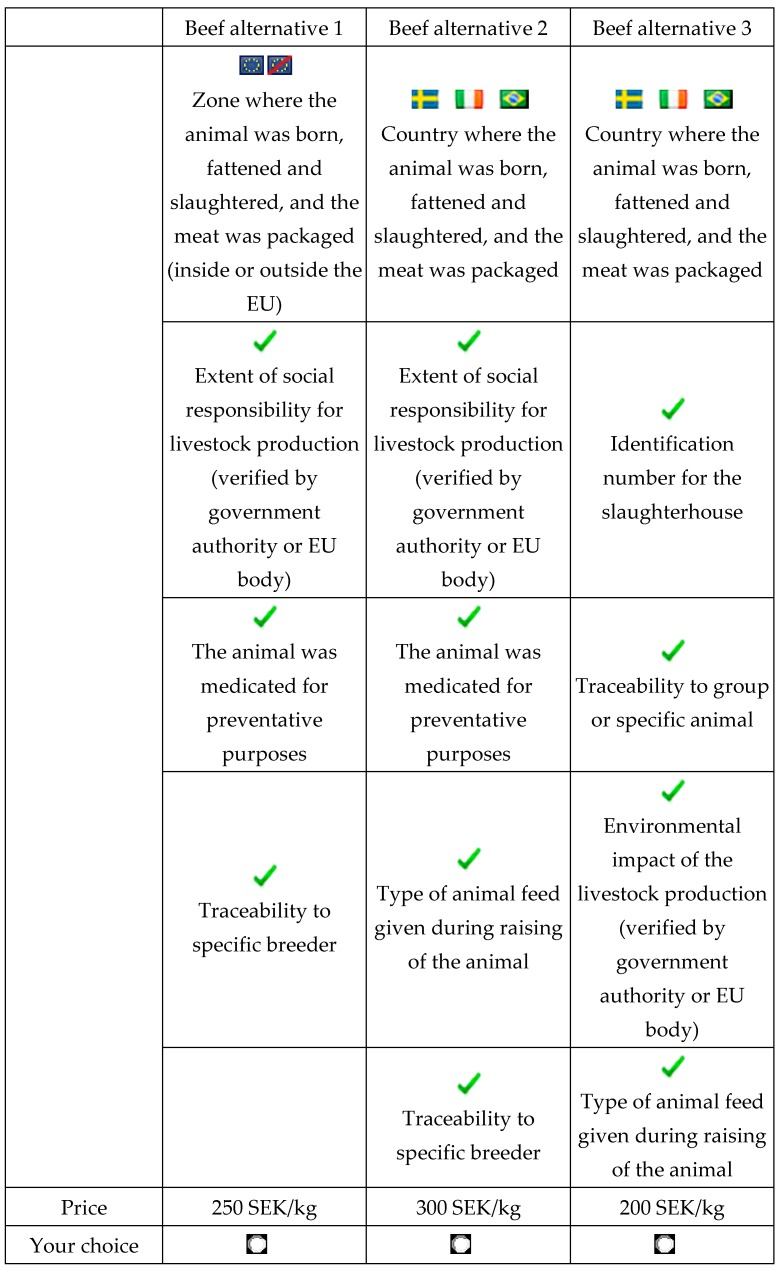
Illustration of a choice set.

An unlabelled choice task approach was taken in the discrete choice experiment (DCE). The heading of each alternative (within choice tasks) was generic (i.e., beef alternative 1, beef alternative 2, etc.), and the only way to discriminate between the alternatives was through the attributes. An example of a choice task used for the DCE is given in Figure 1. The respondents were asked to consider at most six attributes (middle and right-hand concept in Figure 1) and at least four attributes (left-hand concept). In this example, information (labels) referring to reference code, farm animal welfare, organic production, and health impact were not presented. The respondents were initially instructed that the DCE was related to beef products such as minute steak, pepper beef, roast beef, sirloin steak, and tenderloin. The respondents were told to assume that all other mandatory information regarding the choice was always present and that the alternatives presented in each choice task only differed in the attributes presented (Table 3). The food labelling rules are set at the European Union level for all member states, and the general labelling requirements are currently set out in Regulation (EU) 1169/2011. This regulation outlines the mandatory information that must be included on all food product labels, including the product name, ingredient list, use-by date, and any specific instructions or conditions of use.

In each choice task, there were three alternatives. Two alternatives always included the specific COO denomination so as to allow for trade-offs between the remaining attributes presented and the specific origin. These alternatives correspond to existing labelling requirements. This was intended to establish a link to random utility theory and avoid the unfeasibility problem. Then there was one alternative with the EU/non-EU denomination of origin. The design provided a constrained balanced approach (i.e., equal occurrence of each attribute, except for the origin attribute, which was present for each alternative). The relative d-efficiency of the partial profile design was 0.93.

Each respondent was then faced with 22 choice tasks, which, drawing on the extant literature following Bradley and Daly [32], represents a point at which most analysts would expect fatigue effects to have set in. However, more recent findings by Hess, Hensher, and Daly [33], who reviewed and tested for scale differences due to the number of choice tasks in datasets within five stated preference studies from a number of disciplines, while controlling for contexts of familiarity with the market in question, showed an absence of fatigue effects. Similarly, Louviere et al. [34] show that there is little loss of reliability and validity when using larger and more complex choice tasks. In fact, the literature suggests that considerable gains can be achieved by increasing the number of choice tasks per respondent such as the generation of learning effects, which increase model structure reliability and precision [35,36,37]. It has been reported that a similar increase in model precision can be obtained by increasing the number of tasks as by proportionally increasing the number of respondents [36]. In addition, increasing the number of choice tasks has been reported to establish a learning effect whereby respondents have been found to learn to draw finer distinctions between attributes as they progress through the choice tasks. The respondents have thereby been reported to focus on brand or COO (as a proxy for other attributes) over price in the first task, while this effect diminishes in subsequent choice tasks [35,37].

Moreover, a complete heterogeneous design [38], rather than a blocked design, was used to increase the statistical efficiency by providing more variation across respondents and to reduce the problems of scale effects (i.e., variations in preferences due to the block of the design from which data were generated). The heterogeneous design meant that the respondents were randomly assigned one of 100 versions of the full design. On completion of the DCE, the respondents were asked to rate (on a scale from 1 = agree to 5 = disagree) their understanding of the task assigned to them in the DCE. This included three statements referring to (i) the ease of understanding of how to provide responses; (ii) an understanding of the labelling attributes; and (iii) the ability to express what is important concerning the labelling of beef. Furthermore, the respondents were asked (on a scale from 1 = very easy to 5 = very difficult) to rate the perceived difficulty in expressing which type of beef labelling information was important. This was to infer the cognitive burden related to the responses.

Given that the standard information is provided on the label or on the package, which of the following three beef products would you prefer? (The country flags are only illustrative).

Mark your choice by using the buttons, and please bear in mind the price that is associated with your choice:

### 3.3. Statistical Analysis

Random utility theory (RUT) provides a family of probabilistic choice models that describe how choice probabilities relate to changes in choice tasks (i.e., attributes and their levels) and to individual choosers. In accordance with RUT, Equation (1) then describes the probability of individual *n* choosing alternative *i* from the choice task *C_n_* equalling the probability of the systematic ($V_{in}$) and random components ($\varepsilon_{in}$) of the latent unobservable utility associated with alternative *i* for individual *n* being larger than the systematic and random components of all other alternatives competing with alternative *i* within the choice task Equation (2) [39].

(1) $$P\left(i|C_{n}\right)=P\left[\left(V_{in}+\varepsilon_{in}\right)>Max\left(\left(V_{jn}+\varepsilon_{jn}\right)\right)\right]$$

where for all j options in choice set C*_n_*.

The analysis of the DCE data was adapted to the study’s purpose of examining the choice probability for a product alternative with an EU/non-EU denomination of origin conditional upon the depth and content of the consideration set available (i.e., credence quality cues). Therefore, a mixed logit model approach was developed. The model takes the nested nature between choice of denomination (individual preferences) and the (exogenous) explanatory variables into account within mixed effect estimation, thus allowing a random error component so as to capture individual heterogeneity in responses within and across choice sets.

In modelling the nested data structure of *i* persons who completed *j* choice tasks, with each task including *k* choice concepts, the general structure of the mixed logit model used was:

(2) y=Xβ+Zζ+ε

where the *r × n* matrix ***X*** is a representation of the *r* explanatory variables; *β* is a *r × 1* matrix of the parametric coefficients to be estimated on ***X***; the *q × n* matrix ***Z*** is a representation of the *q* random effects; *ζ* is a *q × 1* matrix of the random effect coefficients to be estimated on ***Z*** so that it captures parts of the unobserved heterogeneity of the respondents; and *ε* is the idiosyncratic error of unexplained variance in the dependent variable. However, *y* is specified as a variable that follows a binomial distribution; for each respondent and for each choice concept in each choice task, this dependent variable takes a value of one for all those observations under which a respondent has chosen the EU/non-EU denomination of origin rather than the ‘specific country’ and a value of zero otherwise. The binomial distribution of *y* is consistent with the design of the choice sets. Therefore, we employed a logistic link function

(3) $$g\left(\cdot \right)=ln\left(\frac{p}{1-p}\right)$$

such that the model in Equation (2) became:

(4) g(E(y))=Xβ+Zζ+ε

This type of model is less common in econometrics but is widely used for experimental data obtained, e.g., in crop sciences, medicine, or psychology. In these disciplines, the models are known as ‘linear mixed models’ (e.g., [40]). In contrast to the conditional logit model that is commonly used in the context of choice experiments, our approach has much more flexibly, which allows it to capture unobserved heterogeneity within the data through the random effects ζ.

The model in Equation (4) was estimated using a Restricted Maximum Likelihood (REML) model, as implemented in the lme4 package [40,41] from the R network software [42].

The model specification and the selection of the final model were based on the following steps:

1. The model was set to explain the choice of the dependent variable ‘EU/non-EU origin’ as a function of price level and the number of additional attributes provided as explanatory variables ***X***.
2. Alternative specifications of ***Z*** were estimated as random effects; the selection of the best random effects specification was based on Likelihood Ratio tests for model selection.
3. The model was tested under alternative specifications of the explanatory variables, treating ‘Price level’ and ‘Number of information items provided’ as either discrete or continuous variables or as a combination thereof.
4. In a second set of regressions, the variable containing the number of information attributes was replaced by dummy variables for the actual information categories that were provided.

The marginal effects were computed according to procedures outlined by Fernihough [43], but, with the code provided, they were revised because it simulates only one standard error for all marginal effects. The marginal effects in the present study were averages of the sample marginal effects (rather than average marginal effects) and were computed by multiplying each coefficient $\hat{\beta }$ estimated from Equation (4) by the transformed values from the logistic probability density function of the predicted values [43].

## 4. Results

### 4.1. Consumer Use of Labelling Information

The majority of respondents indicated that they found the response format easy to understand and that they grasped the meaning of the labelling alternatives (Table 4). Furthermore, with respect to the relevance of labelling attributes to judgement and choice, the majority of respondents indicated that they were able to express the importance that they assigned to beef labelling attributes (Table 4). Moreover, the results suggest that the respondents considered the cognitive burden in expressing attribute importance to be low, with almost 50% of respondents reporting the task to be easy or fairly easy (Table 4).

### 4.2. Attribute Information Search

**Figure 2:**
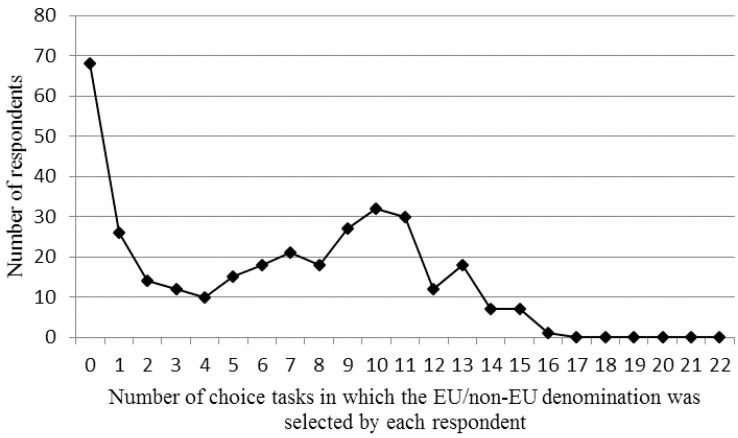
Choice frequency for the EU/non-EU origin denomination within the choice experiment. Each respondent saw 22 choice sets.

There were *i* = 336 respondents who completed *j* = 22 choice tasks, with each task including *k* = 3 choice concepts, leading to *n* = 22,176 observations. In the DCE, 68 respondents (20.2%) never chose an alternative with the EU/non-EU denomination. This left 268 respondents who chose the EU/non-EU labelled denomination in at least one choice set. The distribution of the number of times that each respondent selected the EU/non-EU denomination within the DCE is presented in Figure 2. Among the respondents who selected it at least once, there was a minority (*n* = 52) with less frequent use (maximum three selections) of this alternative, whereas the average was 6.2 (SD = 4.7). In total, the EU/non-EU alternative as denomination of origin was selected 2094 times.

### 4.3. Amount of Information Sought

**Figure 3:**
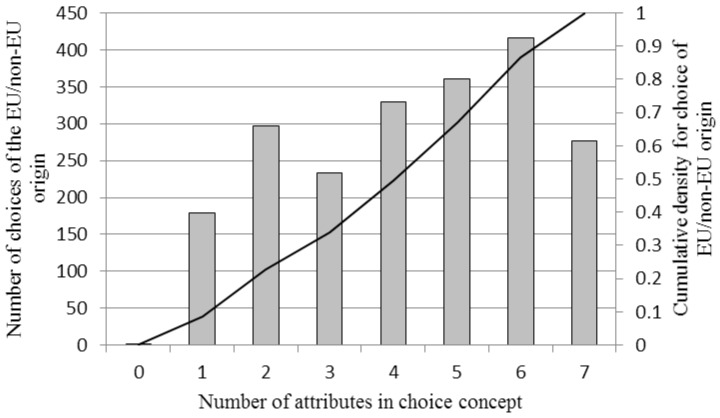
Histogram and cumulative density function for the number of choices of the EU/non-EU origin alternative by the number of extrinsic attributes in the choice experiment.

**Figure 4:**
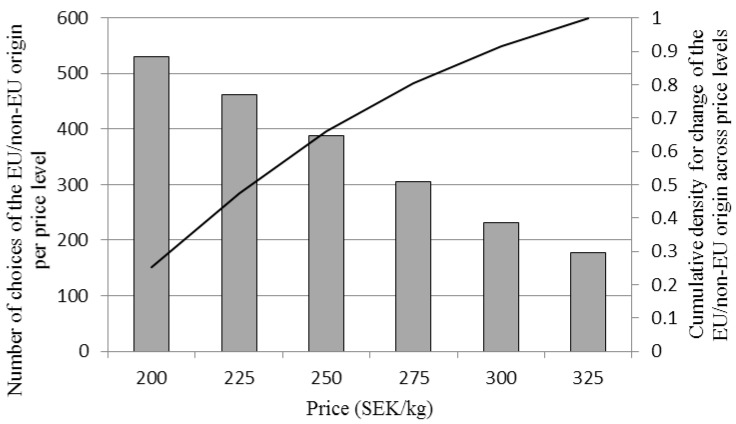
Histogram and cumulative density function for the levels of the price attribute when the EU/non-EU origin alternative was selected in the choice experiment.

The relationship between the number of extrinsic attributes present in the choice concept when selecting the EU/non-EU denomination is presented in Figure 3 and that between the levels of the price attribute and the selection of the EU/non-EU denomination in Figure 4. The probability of selecting the EU/non-EU denomination was increasingly related to the number of extrinsic attributes present in each choice concept up to the level of six additional attributes. Furthermore, the probability of selecting the EU/non-EU denomination was highest for lower levels of the price attributes and then decreased as the price level increased.

### 4.4. Content and Compensatory Effects Related to Origin

When estimating the Restricted Maximum Likelihood (REML) model for explaining the choice of the EU/non-EU denomination as a function of price level and the number of extrinsic attributes, it was found that, in all model specifications, the model without random effects was rejected based on Akaike information criterion (AIC) and likelihood ratio tests. Furthermore, when assessing the alternative random effect specifications, it emerged that models with random effects for individual respondents and concepts performed best according to the AIC and likelihood ratio test criteria, respectively.

As Table 5 (marginal effects) shows, the effect of a one-unit change in price on the log of odds of choosing beef with a EU/non-EU denomination compared with a product with a specific COO denomination was negative, whereas the effect of adding information through the provision of additional extrinsic attributes was positive. In this calculation, dummy variables for price levels (base level was set at 200 SEK/kg) and the number of extrinsic attributes (base level was set at zero) were used. The negative estimates for higher levels of the price attribute and the estimates for the information provision confirm the indications in Figure 3 and Figure 4 of a declining propensity to select the EU/non-EU denomination at higher price levels. The results also indicate that a positive information effect exists already for one additional labelling attribute and that the marginal effect then declines for the provision of two to three additional attributes but increases again and reaches its maximum at six additional attributes, after which it declines.

In contrast, the null hypothesis that all fixed effects on price are jointly zero was strongly rejected (Probability (>χ^2^) < 0.001)) by a Wald test of the restricted model against the unrestricted model. Furthermore, when testing the null hypothesis of equality for any two pairs of estimated coefficients on the price levels, this null hypothesis was rejected throughout (*p* < 0.01). This implies that price changes have an effect on choice decisions and that there is no reason to expect a price range within which a change in the product price would not matter.

When testing for the joint equality of the estimated coefficients on fixed effects for the number of information attributes provided, the null hypothesis of equality of any two coefficients was rejected for all pairs of coefficients, except for the case of six versus seven information attributes (Pr (>χ^2^) = 0.23)).

Furthermore, we tested for interaction effects between the total number of extrinsic attributes present in the choice concept and the price level in order to identify interdependencies between information provision and price. The results from estimation of the REML model are presented in Table 6. It was not possible to estimate other combinations of interaction effects due to singularities. Moreover, it should be noted that the measuring scale for the information variables differs between Table 5 (nominal) and Table 6 (metric). In Table 5, the count of information attributes provided during the experiment is added as a dummy, while Table 6 refers to the continuous number of information attributes.

Interestingly, the results in Table 6 suggest that the provision of more cues alone did not significantly increase the probability of a respondent choosing the EU/non-EU denomination. On the other hand, an increasing price level of a beef product with the EU/non-EU denomination alone was sufficient to decrease the choice probability, while the null hypothesis that all fixed effects on price are jointly zero was again strongly rejected.

However, the joint effect of the price variable and the number of extrinsic attributes was found to be positive and significant, although the significance level for the price level of SEK 225 per kg was just below the five percent threshold (Table 6). Taken together, this suggests that a higher price level and more information give a slightly positive effect, but the increasing marginal effect of this is much smaller than the decreasing negative marginal effect on price. Thus, even though there was a partial positive effect of higher price and more information on the likelihood of selecting the EU/non-EU origin denomination, it is most likely that this effect would be over-compensated for by the negative price effect.

The extent to which each labelling attribute influenced the choice of the EU/non-EU denomination (i.e., the content part of information processing in accordance with [13]) is reported in Table 7. The REML model was then re-estimated with each credence attribute coded as a dummy variable. This part of the analysis addressed the compensatory qualities of each credence attribute in relation to the zone of origin denomination. It was found that the extent of good animal welfare and information about whether the animal was medicated for preventative purposes had the highest marginal effects. The results from the model also suggested that information about organic production and traceability to a group or a specific animal had an intermediate influence over the respondents’ choice. The type of animal feed used during production and traceability to either a specific slaughterhouse or a specific breeder had the lowest positive effect on the choice of the EU/non-EU denomination but were still significant factors. In this context, the null hypothesis that all fixed effects on price are jointly zero was again strongly rejected, as was the pair-wise null hypothesis of equal coefficients on discrete price levels. This implies that there was no price range statistically less important for the choice decision. Equality testing for the credence attributes was not performed since any labelling attributes or interactions between these should be allowed to have the same coefficient by chance.

Turning to a comparison between the REML models for the amount of information provided (i.e., Table 5) and the content of information provided (i.e., Table 7), a likelihood ratio test of (two times) the difference of the log-likelihoods from both models confirmed that the specification in Table 7 fitted the data significantly better (χ^2^ = 123.32 *degrees of freedom (df)* = 4, Pr (>χ^2^) < 0.001). In this standard test, the model in Table 5 (df = 15) served as the ‘null’ model and the model in Table 7 (*df* = 19) as the ‘alternative’ model. The underlying null hypothesis of this test was that the model with more informative parameters does not fit the data significantly better (according to the log-likelihood) than the model with fewer informative parameters. In the test, this hypothesis was rejected.

## 5. Discussion

Existing data show that country images (origin) provide cognitive, ethical, and moral meaning to consumers making comparisons of domestic and imported food [44]. These COO images have a direct affective effect (i.e., sense of belonging) on consumers’ purchasing decisions [19,45]). A vast body of literature has reported that consumers in many countries have preferences for domestic beef (e.g., [2,46,47]). This line of research has provided evidence on the importance of country or, more locally, specific origin denomination and, when the methodology has allowed, on the relative importance of origin versus other attributes included in the studies. However, such research has typically provided less information about the drivers for alternative levels of the origin used within each study.

Despite the importance attributed to a specific COO denomination, the possibility that consumers would be willing to choose beef with an alternative denomination of origin when such is available still cannot be ruled out. A recent study by Tonsor, Schroeder, and Lusk [48] reported that US consumers did not differentiate their valuation of meat products with ‘Product of North America’ and ‘Product of the United States’ labels, whereas products carrying the label ‘Product of Canada, Mexico, and US’ were the least preferred. The results from the present study confirm that a choice of the broader denomination instead of the country-specific denomination is possible: 268 out of 336 respondents (79.8%) chose the broader denomination instead of the country-specific denomination in, on average, 6.2 out of 22 choice sets (28%).

### 5.1. Amount of Information Sought

The analysis examined how the choice of EU/non-EU origin depended on the number of other credence attributes together with the price information. The estimated marginal effects related to the number of other credence attributes presented provided somewhat mixed results. The largest choice likelihood occurred with as many as six additional attributes. Therefore, when weighted against labelling with a specific COO, the EU/non-EU origin alternative required a larger set of additional information to compensate for and qualify the decision. However, the likelihood function was asymmetrical, with the provision of only one additional credence attribute as the second most decisive level of information. Verbeke and Ward [24] and Verbeke and Roosen [49] found weak consumer interest in meat labelling information. Hence, having just one additional cue functioned just as well as having more. One reason for the stronger requirement for more information found in the present study may be that the choice of the EU/non-EU denomination triggered a more analytical evaluation. This seems plausible, as respondents indicated that their perceived level of difficulty with the choice format was low and that they were able to understand the meaning of the attributes quite well. In a more contextual setting, consumers would face a broader set of labelling information. Further testing in real purchasing occasions would bring an understanding of the compensatory role of credence labelling information for consumer acceptance of the EU/non-EU denomination.

Furthermore, the probability of choosing the EU/non-EU origin denomination decreased in relation to increasing price for the beef concepts. This effect of price corroborated results by Mesías et al. [50] and reflected that beef with this origin label is considered a normal good.

Another finding concerned the joint influence of price and the number of other credence attributes for the joint probability of choosing the EU/non-EU denomination of origin. The provision of information, irrespective of extent, was found to have a much lower influence than price. Keeping price constant while increasing the extent of other credence information had no significant effect on choice probabilities, while the opposite significantly reduced the choice probabilities. The existence of a nested effect of price and scope of labelling information has received little attention within research on food decision making. This suggests that the preferences between the price attribute and the other attributes may not be weakly separable, meaning that the underlying utility function would not be linear in its arguments, as is typically assumed, but rarely asserted, in the mainstream research using DCE to estimate willingness-to-pay for food quality attributes.

### 5.2. Content and Compensatory Effects Related to Origin

Lastly, this study examined the importance of different types of information so as to identify the major drivers for the probability of choosing the EU/non-EU denomination rather than the specific COO denomination. Bernués et al. [2] noted that information about production systems and quality control constitutes credence cues, which can be transformed into search attributes to guide the evaluation of concerns by the consumer. It has been predicted that future developments in the production and consumption of beef will focus on environmental protection, animal welfare, health benefits related to nutrition, and aspects relating to responsibility [51].

Findings concerning attributes related to production systems showed that labelling information on the extent of good animal welfare during production was the most decisive attribute to drive the choice of the EU/non-EU denomination of origin. Information about whether or not the animal was medicated for preventative purposes was also of high importance for consumers’ judgement and the choice of the origin denomination. The use of preventive medication such as antibiotics is in itself a typical indicator of animal welfare problems [52]. However, such information has not yet been provided to Swedish meat purchasers, as, by national law, medication is only permitted based on confirmed disease cases. Notably, the type of feed given when raising the animals was of the lowest importance among the factors to increase choice probability. This aspect is of relevance in relation to the importance given to animal welfare, as there is a relationship between rearing systems and feed use.

Interestingly, the attributes related to traceability were of the least decisive importance. This could be taken to indicate the passive nature of this type of labelling information, as such details are needed just in case of adverse outcomes. Among the cues related to quality control, traceability to group or specific animal had a higher influence on choice probabilities. As the additional attributes related to a specific slaughterhouse, which is mandatory information, or to a specific breeder, this result means that consumers choosing the broader EU/non-EU denomination gave priority to greater depth in their information search. Interestingly, the marginal choice effect for the reference code attribute, which is also mandatory, was similar to the effect of traceability to a specific group or animal. The reference code itself is of little use in guiding the consumer with respect to information content. The important information found here may mean that consumers assign a value to the objective nature of this attribute.

Together, these findings corroborate findings in earlier studies that indicate that Swedish consumers place high importance on more explicit animal welfare aspects in livestock production (e.g., [53]). Furthermore, information about organic production, the extent of environmental impact from livestock production, and the extent of social responsibility was given an intermediate position of influence on the choice probability. Information about the impact on health from the consumption of beef was at the same level as information on environmental impacts. This is interesting as it suggests that individuals have weighed a direct effect (health) equally with an indirect effect (environment).

## 6. Conclusions

For the food industry, a decision on the particular labelling of origin information to provide this to final consumers may have implications for competitive advantage. Firms within food supply chains typically operate private systems for traceability, transparency, and quality assurance. Further obligations set by mandatory labelling requirements for identity or product segregation are costly, with the potential to distort investments and marketing incentives in relation to markets or products with fewer such obligations. Hence, an EU/non-EU label of origin for meat could reduce the costs of segregation and identity preservation, increase the mobility of meat produced within the EU, and affect trade, leading to potential consumer price decreases.

This study showed that adopting a more general EU/non-EU label of origin, instead of today’s mandatory label of specific COO, would require priority to be given to information depth (i.e., the amount of information sought) and content (i.e., the type of information examined). Regarding the depth of information, we found that relatively many informational items were used by consumers as a basis for choosing the EU/non-EU label of origin. We also found that consumers in such decisions considered the joint influence of price and depth of information, with the price being the overwhelming aspect influencing consumer behaviour. As the provision of information is costly from the perspective of the industry, this means that the EU/non-EU origin label would be more useful for products in the lower range of the quality span. Regarding the content of information, we found that Swedish consumers in this case would give priority to information relating to animal welfare. Far-reaching information on traceability to a specific group or animal was also found to be of high importance.

## Figures and Tables

**Table 1:** Characteristics of the sample, % (*n* = 336).

Variable	Category	Proportion
Age ^a^	18–34	29.9
	35–49	35.1
	50–75	45.0
Gender ^b^	Male	54.0
	Female	46.0
Household income (gross monthly)	≤SEK 20,000	11.3
	SEK 20–40,000	30.2
	SEK 40,001–60,000	31.5
	≥SEK 60,000	14.1
	No information	12.9
Household size	1 person	24.8
	2 persons	42.4
	3–4 persons	25.4
	≥4 persons	7.4
Location of dwelling	Large city area (≥150,000)	34.4
	Medium size city area (50–150,000)	30.5
	Rural or small city area (≤50,000)	34.7
	No information	0.3
Level of highest education	Primary school	5.8
	Secondary school	36.3
	College or equiv. (≤3 years)	18.0
	University or equiv. (>3 years)	28.6
	Other higher education	10.9
	Other	0.3

^a^ The corresponding values for the Swedish population (2012) are: 30.7% (18–34); 29.9% (35–49); and 41.2% (50–75) (Statistics Sweden, 2012). ^b^ Statistics Sweden (2012) reports that 49.5% of the Swedish population aged between 18 and 75 years is female.

**Table 2:** Respondents’ stated use of labelling information when buying beef as a percentage.

Statement 1	Alternative	Proportion
To what extent would you say that you look at the labelling information (on the package) when you buy beef today?	I look at all	17.4
	I look at most	36.0
	I look at some, but not all	32.5
	I look at just a few	11.6
	I do not look at it	2.6

**Table 3:** The labelling attributes used and their levels in the choice experiment.

Attribute 1	Level
Origin	Label for specific country of origin available; or label for geographical zone of origin (beef labelled with origin as either inside or outside the EU) available
Reference code	Label present on package/not present
Traceability to specific slaughterhouse	Label present on package/not present
Traceability to group or specific animal	Label present on package/not present
Traceability to specific breeder	Label present on package/not present
Extent of good animal welfare for livestock production ^a^	Label present on package/not present
Health impact from consumption of beef ^a^	Label present on package/not present
Extent of social responsibility for livestock production ^a^	Label present on package/not present
The animal was medicated for preventative purposes	Label present on package/not present
Type of animal feed given during raising the animal	Label present on package/not present
Price ^b^ (SEK) per kilogram	200, 225, 250, 275, 300, 325

^a^ Verified by government authority or EU body. ^b^ At the time of the survey 1 SEK = 0.11 EUR or 0.14 USD.

**Table 4:** Respondents’ evaluation of the response formats as percentages.

Statement	Alternative	Proportion
It was easy to understand how I should provide my choices	Disagree	6.1
	Partly disagree	18.6
	Neutral (neither disagree nor agree)	21.9
	Partly agree	24.4
	Agree	28.9
I understood the meaning of the labelling alternatives	Disagree	2.6
	Partly disagree	10.9
	Neutral (neither disagree nor agree)	21.9
	Partly agree	37.6
	Agree	27.0
I was able to express what was important for me concerning beef labelling	Disagree	2.9
	Partly disagree	10.6
	Neutral (neither disagree nor agree)	20.3
	Partly agree	41.2
	Agree	25.1
How did you find expressing which type of beef labelling information was important to you?	Very easy	10.3
	Fairly easy	39.5
	Neither easy nor difficult	24.4
	Fairly difficult	23.8
	Very difficult	1.9

**Table 5:** Restricted Maximum Likelihood estimates.

Parameter Estimates	Estimate	Standard Error	Standard Score (*z*)	Probability (>|z|)	Marginal Effects	Standard Error
(Intercept)	−5.834	0.724	−8.062	<0.001		
Price level ^1^ = 2	−0.151	0.072	−2.097	0.036	−0.012	0.012
Price level = 3	−0.374	0.075	−4.993	<0.001	−0.029	0.024
Price level = 4	−0.682	0.079	−8.601	<0.001	−0.053	0.042
Price level = 5	−1.026	0.086	−11.938	<0.001	−0.080	0.063
Price level = 6	−1.296	0.093	−13.885	<0.001	−0.101	0.079
Info = 1	3.743	0.722	5.181	<0.001	0.292	0.229
Info = 2	3.309	0.720	4.593	<0.001	0.258	0.212
Info = 3	3.124	0.721	4.332	<0.001	0.244	0.200
Info = 4	3.716	0.720	5.157	<0.001	0.289	0.234
Info = 5	3.968	0.720	5.509	<0.001	0.309	0.246
Info = 6	4.182	0.720	5.809	<0.001	0.326	0.261
Info = 7	4.077	0.721	5.654	<0.001	0.318	0.254
						
Random effects	Groups	Name	Variance	Standard Deviation.		
	Respondents	(Intercept)	1.2809	1.1318		
	Alternative	(Intercept)	0.0073	0.0855		
						
	Akaike Information Criterion	Bayesian Information Criterion	Log Likelihood	Deviance		
	12,530	12,650	−6250	12,500		
Number of observations	22,176					

^1^ Price levels 2 (225 SEK/kg) to 6 (325 SEK/kg). Price 1 (base level) was 200 SEK/kg. At the time of the survey 1 SEK = 0.11 EUR or 0.14 USD. ‘Info’ refers to the number of additional credence attributes (beyond origin and price) within the choice concept when EU/non-EU origin was selected. Underline: Present the two components for the random effects estimation in multilevel modelling.

**Table 6:** Restricted Maximum Likelihood estimates.

Parameter Estimates	Estimate	Standard Error	Standard Score (*z*)	Probability (>|z|)	Marginal Effects	Standard Error
(Intercept)	−2.249	0.140	−16.006	<0.001		
Price level^1^ = 2	−0.440	0.170	−2.591	0.010	−0.035	0.030
Price level = 3	−0.716	0.174	−4.112	<0.001	−0.057	0.044
Price level = 4	−1.202	0.191	−6.306	<0.001	−0.095	0.070
Price level = 5	−1.611	0.212	−7.611	<0.001	−0.128	0.094
Price level = 6	−1.780	0.227	−7.929	<0.001	−0.143	0.104
Info	0.023	0.026	0.902	0.367	0.002	0.003
Price level = 2 × Info	0.071	0.037	1.907	0.057	0.006	0.0053
Price level = 3 × Info	0.083	0.038	2.188	0.029	0.007	0.006
Price level = 4 × Info	0.122	0.041	3.008	0.003	0.010	0.008
Price level = 5 × Info	0.139	0.044	3.180	0.002	0.011	0.009
Price level = 6 × Info	0.123	0.048	2.585	0.010	0.010	0.008
						
Random effects	Groups	Name	Variance	Std.Dev.		
	Respondents	(Intercept)	1.247	1.117		
	Alternative	(Intercept)	0.008	0.090		
						
	Akaike Information Criterion	Bayesian Information Criterion	Log Likelihood	Deviance		
	12,812	12,924	−6392	12,784		
Number of observations	22,176					

^1^ Price levels 2 (225 SEK/kg) to 6 (325 SEK/kg). Price 1 (base level) was 200 SEK/kg. ‘Info’ refers to the set of credence attributes (beyond origin and price) within the choice concept when EU/non-EU origin was selected. Underline: Present the two components for the random effects estimation in multilevel modelling.

**Table 7:** Discrete price level with each informational attribute treated as a dummy variable.

Parameter Estimates	Estimate	Standard Error	Standard Score (*z*)	Probability (>|z|)	Marginal Effects	Standard Error
(Intercept)	−3.237	0.113	−28.631	<0.001		
Price level^1^ = 2	−0.145	0.072	−2.000	0.046	−0.011	0.011
Price level = 3	−0.385	0.076	−5.099	<0.001	−0.030	0.025
Price level = 4	−0.713	0.080	−8.921	<0.001	−0.055	0.044
Price level = 5	−1.0636	0.087	−12.279	<0.001	−0.082	0.066
Price level = 6	−1.308	0.094	−13.924	<0.001	−0.101	0.081
Reference code	0.303	0.051	5.971	<0.001	0.023	0.019
Trace. to spec. slaughterhouse	0.211	0.051	4.155	<0.001	0.016	0.013
Trace. to group/spec. animal	0.290	0.051	5.710	<0.001	0.022	0.018
Trace. to spec. breeder	0.216	0.051	4.255	<0.001	0.017	0.014
Animal welfare	0.419	0.050	8.351	<0.001	0.032	0.026
Medicated prevent. purposes	0.366	0.050	7.249	<0.001	0.028	0.023
Organic production	0.294	0.050	5.846	<0.001	0.023	0.018
Environmental impact	0.244	0.050	4.817	<0.001	0.019	0.016
Health impact	0.248	0.051	4.861	<0.001	0.019	0.016
Extent social responsibility	0.284	0.051	5.604	<0.001	0.022	0.018
Type of animal feed	0.209	0.051	4.115	<0.001	0.016	0.014
						
Random effects	Groups	Name	Variance	Std.Dev.		
	Respondents	(Intercept)	1.321	1.149		
	Alternative	(Intercept)	0.009	0.097		
						
	Akaike Information Criterion	Bayesian Information Criterion	Log Likelihood	Deviance		
	12,415	12,567	−6188	12,377		
Number of observations	22,176					

^1^ Price levels 2 (225 SEK/kg) to 6 (325 SEK/kg). Price 1 (base level) was 200 SEK/kg. Underline: Present the two components for the random effects estimation in multilevel modelling.
